# The negative impact of spasticity on the health-related quality of life of stroke survivors: a longitudinal cohort study

**DOI:** 10.1186/s12955-015-0340-3

**Published:** 2015-09-29

**Authors:** Patrick J. Gillard, Heidi Sucharew, Dawn Kleindorfer, Samir Belagaje, Sepideh Varon, Kathleen Alwell, Charles J. Moomaw, Daniel Woo, Pooja Khatri, Matthew L. Flaherty, Opeolu Adeoye, Simona Ferioli, Brett Kissela

**Affiliations:** Department of Global Health Outcomes Strategy and Research, Allergan, Inc., 2525 Dupont Drive, Irvine, California, 92612-1531 USA; Division of Biostatistics and Epidemiology, Cincinnati Children’s Hospital Medical Center, 3333 Burnet Avenue, MLC 5041, Cincinnati, OH 45229-3039 USA; Department of Neurology and Rehabilitation Medicine, University of Cincinnati, 260 Stetson St., Suite 2300, PO Box 670525, Cincinnati, OH 45267-0525 USA; Departments of Neurology and Rehabilitation Medicine, Emory University School of Medicine, Faculty Office Building, Rm# 375, 80 Jesse Hill Jr Dr. SE, Atlanta, GA 30303 USA; Department of Neurology and Rehabilitation Medicine, University of Cincinnati, Division of Neurocritical Care, University of Cincinnati, 231 Albert Sabin Way, ML 0769, Cincinnati, OH 45267 USA; Department of Emergency Medicine and Neurosurgery, University of Cincinnati, 231 Albert Sabin Way, ML 0769, Cincinnati, OH 45267 USA; Department of Neurology and Rehabilitation Medicine, University of Cincinnati, University Hospital Medical Arts Building, 222 Piedmont Avenue, Suite 3200, Cincinnati, OH 45219 USA

**Keywords:** Stroke, spasticity, quality of life, stroke rehabilitation

## Abstract

**Background:**

Spasticity often leads to symptomatic and functional problems that can cause disability for stroke survivors. We studied whether spasticity has a negative impact on health-related quality of life (HRQoL).

**Methods:**

As part of the Greater Cincinnati/Northern Kentucky Stroke Study (NCT00642213), 460 ischemic stroke patients were interviewed during hospitalization and then followed over time. HRQoL was measured by the Physical Component Summary (PCS) and Mental Component Summary (MCS) scores of the Short Form-12 (SF-12), EuroQol-5 dimension (EQ-5D), and Stroke-Specific Quality of Life (SSQOL) instruments, with lower scores indicating worse health. HRQoL differences between stroke survivors with and without spasticity were compared, adjusting for age, race, stroke severity, pre-stroke function, and comorbidities.

**Results:**

Of the 460 ischemic stroke patients, 328 had spasticity data available 3 months after their stroke (mean age of 66 years, 49 % were female, and 26 % were black). Of these patients, 54 (16 %) reported having spasticity. Three months following their stroke, patients who reported spasticity had lower mean scores on the PCS (29.6 ± 1.4 vs 37.3 ± 0.6; *P* < .001), EQ-5D (0.59 ± 0.03 vs 0.71 ± 0.01; *P* < .001), and SSQOL (3.57 ± 0.08 versus 3.78 ± 0.03; *P* = .03) compared with patients who did not report spasticity. Lower HRQoL scores were also observed at the 1-year (PCS, EQ-5D, and SSQOL) and 2-year (EQ-5D and SSQOL) interviews in those with spasticity compared with those without spasticity.

**Conclusions:**

Statistically and clinically meaningful differences in HRQoL exist between stroke survivors with and without spasticity.

## Background

Each year, 15 million people worldwide, including approximately 795,000 Americans, experience a new or recurrent stroke [1, 2]. Stroke ranks fourth among all causes of death and is a leading cause of serious, long-term disability in the United States [2]. Spasticity, a sensorimotor disorder characterized by a velocity-dependent increase in muscle tone with exaggerated tendon jerks, has been estimated to occur in up to 46 % of patients 12 months after stroke [3–5].

Although spasticity can be regional or generalized, stroke survivors commonly experience focal spasticity in their upper and/or lower limbs. In an analysis by Urban and colleagues, PSS in the upper and/or lower limbs occurred in approximately 43 % of patients 6 months after stroke among patients with clinical signs of central paresis [4]. Stroke survivors with spasticity often experience secondary limb deformities, physical disability, and pain that limits their ability to perform basic activities of daily living, such as holding or picking up objects, self-care, and ambulation. Stroke survivors with spasticity also often suffer from psychological and emotional issues, such as depression and poor self-image [6]. As a result, spasticity has been hypothesized to have a significant negative impact on the health-related quality of life (HRQoL) of stroke survivors. To date, the published evidence to support this negative association is limited [7]. Most existing supportive information comes from clinical trials that are short in duration and not generalizable to the United States population.

Given the extent to which spasticity is present in stroke survivors and the paucity of data examining the impact of spasticity on stroke survivors’ HRQoL, we utilized a longitudinal cohort study of stroke survivors to examine the impact of spasticity on HRQoL.

## Methods

### Study design

This work was undertaken through the Greater Cincinnati/Northern Kentucky Stroke Study (GCNKSS), a 5-county population-based study designed to characterize the stroke population in the greater Cincinnati metropolitan area and to determine the regional incidence of stroke and mortality. GCNKSS was approved by the institutional review boards at all participating institutions. Study methods have been described elsewhere [8].

### Study participants

As part of the 2005 cohort of the GCNKSS, a group of ischemic stroke patients was prospectively identified from the larger cohort in order to examine poststroke clinical and HRQoL outcomes. Ischemic stroke patients were identified as having had an ischemic stroke based on the clinical criteria adapted from the Classification of Neurological Disorders III and epidemiological studies of stroke for cerebral ischemia and a review of medical records, including all available neuroimaging. All adult (≥18 years of age) ischemic stroke patients who presented during 2005 at any of the 17 hospitals in the study area were eligible for study enrollment if they resided in the 5 study area counties.

After a potential patient was identified, the patient’s treating physician was contacted for permission to approach the individual for informed consent. Written informed consent was obtained either from the patient or from a proxy for patients who were unable to supply reliable information or were unresponsive, aphasic, or confused. Patients unable to be interviewed or provide informed consent (independently or by proxy) and patients diagnosed with a terminal illness prior to stroke with a life expectancy of <12 months were not enrolled in the study.

For each enrolled patient, detailed medical record abstractions were undertaken by trained nurses to capture key information about demographics, pre-stroke level of functioning, past medical history, and testing and laboratory results. Stroke severity was estimated from the medical record using the retrospective National Institutes of Health Stroke Scale (rNIHSS) score [9]. To assess functional and HRQoL outcomes, a detailed in-person interview was performed in the early poststroke period, and phone interviews were conducted at 3 months, 1 year, and 2 years poststroke in surviving cohort members. Interviews were conducted by trained study nurses. Comorbidities and other medical conditions were first identified by retrospective review of hospital charts during the 3-month poststroke period and by phone interview at 3 months, 1 year, and 2 years. Details of the interviews have been published previously [10].

As part of the 3-month and annual interviews, each surviving poststroke patient was asked if he or she had experienced spasticity following his or her stroke (e.g., “Did you have any spasticity following your stroke?”). The interviewer explained the term “spasticity” as if the patient did not recognize the term. For this analysis, if the patient answered “yes” to this question, he or she was classified as a poststroke patient with spasticity. A patient who answered “no” to the spasticity question was classified as a poststroke patient without spasticity. At different time points (3 months, 1 year, and 2 years), poststroke patients would be reclassified as with or without spasticity depending on their answer to the same question.

### Outcome measures

Three instruments were used to assess HRQoL. As part of each poststroke interview, the interviewer administered the EuroQol-5 dimension (EQ-5D), the Short Form-12 (SF-12), and the Stroke-Specific Quality of Life (SSQOL) instruments.

The EQ-5D (3 levels) is a widely used preference-based generic HRQoL measure that consists of 2 components, the self-classifier and the visual analog scale. The self-classifier (the section of the EQ-5D used in this analysis) provides a simple method for capturing self-reported descriptions of health problems according to a 5-dimension classification system. The 5 dimensions include mobility, self-care, usual activities, pain/discomfort, and anxiety/depression. Each dimension comprises the following 3 levels: (1) no problem, (2) some or moderate problems, and (3) severe or extreme problems. Scores for the 5 dimensions were converted into a utility index that ranges between −0.11 and 1 (United States–weighted) by applying the scores from value sets (i.e., preference weights) elicited from the United States population [11, 12]. A maximum score of 1 indicates perfect health, whereas a score of 0 represents death. A negative score is indicative of health states worse than death (i.e., an individual would prefer death versus being in these health states). There is evidence for the validity of the EQ-5D in the stroke population [13].

The SF-12 is also a widely used generic HRQoL instrument derived from the Short Form-36. The SF-12 includes 12 questions that represent the following 8 health domains: Physical Functioning, Role Limitations due to Physical Health, Bodily Pain, General Health, Vitality, Social Functioning, Role Limitations due to Emotional Problems, and Mental Health. Weighted scores from the 8 health domains were aggregated into the Physical Component Summary (PCS) and the Mental Component Summary (MCS) scores. Scores were normalized for age and sex, resulting in SF-12 scores centered around 50, reflecting the average domain scores of the United States population. Scores >50 indicate better HRQoL, whereas scores <50 denote worse HRQoL [14]. The SF-12 has been shown to be valid in the stroke population.

Unlike the EQ-5D and the SF-12, the SSQOL is a stroke-specific HRQoL instrument that has been validated for telephone data gathering [15]. Although the SSQOL cannot measure HRQoL across populations and across diseases, it may capture important disease-specific information not captured by the generic instruments. A reduced version of the SSQOL consists of 35 items that measure the following 7 health domains: Energy, Role Function, Language, Physical Function, Mood, Thinking, and Vision [16]. Each item is measured on a 5-point Likert scale using 1 of 3 different response sets (e.g. “no trouble at all” to “couldn’t do it at all” for task-oriented items). The overall score was computed as an unweighted average of the domain scores, with higher scores indicating better HRQoL. Although this shortened form of the stroke-specific instrument has not been published, other shortened forms of the stroke-specific instrument have been published [17].

### Statistical analysis

Demographic and baseline clinical characteristics of the stroke cohort were assessed using descriptive statistics and were compared between stroke survivors with and without 3-month spasticity using 2-sample *t*-tests for age; Chi-square test for sex, race, marital status, employment status, smoking status, education, and baseline comorbid conditions; Wilcoxon rank sum test for body mass index, modified Rankin Scale (mRS), lesion volume, and rNIHSS; and Fisher’s exact test for residence status at admission. A cross-sectional approach was used to evaluate differences in mean HRQoL instrument scores between stroke patients with and without spasticity at each time point (3 months, 1 year, and 2 years) using multivariable linear regression models, adjusting for age, race, stroke severity (rNIHSS), pre-stroke function (mRS), and time-specific comorbidities. The SF-12 was not collected at the 2 year follow-up by design to limit the length of the interview questionnaire. As such, analyses using the SF-12 are limited to 3 months and 1 year follow-up. This was followed by linear regression models for longitudinal measures of HRQoL in association with spasticity using generalized estimating equations to account for repeated measurements. This modeling approach accommodates missing data and allows for the use of all data points available to estimate a model, potentially minimizing bias from missing data [18]. Spasticity status was included in the models as a time-varying independent variable. Age, race, rNIHSS, and pre-stroke mRS were included as time-invariant covariates. Comorbidities of cardiovascular disease, diabetes, depression, seizure, and infection in the prior month were included as time-varying covariates. Analyses were conducted using SAS software (version 9.3; SAS Institute Inc., Cary, NC).

## Results

A total of 460 ischemic stroke patients were enrolled in the 2005 cohort. The mean (± standard deviation [SD]) age was 67 ± 14 years, 48 % were female, and 25 % were black. At 3 months poststroke, 328 had spasticity data available and 54 (16 %) reported having spasticity. At 3 months, the mean age of patients with spasticity was lower than those who did not report spasticity (60 vs 67 years), a higher proportion of patients with spasticity was black (39 % vs 23 %), and a higher proportion was on disability (22 % vs 7 %) (Table 1). Employment status at 3 months poststroke differed significantly between patients with spatisticy and patients without spasticity with a higher proportion employed (33 % vs 29 %) and on disability (22 % vs 7 %) and a lower proportion unemployed (44 % vs 64 %) for those with spasticity compared to those without spasticity (P < .01). In addition, a higher proportion of patients with spasticity had a seizure history (15 % vs 4 %) and patients with spasticity had a more severe stroke (median rNIHSS 6 vs 4) compared with those patients without spasticity (Table 2). At 1 year poststroke, 243 patients had spasticity data available and 43 (18 %) reported having spasticity. Thirty eight (16 %) patients changed spasticity status [19 (8 %) present to absent; 19 (8 %) absent to present]. At 2 years poststroke, 187 patients had spasticity data available and 31 (17 %) reported having spasticity. Twenty five (13 %) patients changed spasticity status [14 (7 %) present to absent; 11 (6 %) absent to present]. Over the 3 poststroke time points, 365 patients had spasticity data available at some point during follow-up, and those patients were included in the longitudinal HRQoL models. Of the 365 patients included in the longitudinal models, 91 patients reported having spasticity at least once.

**Table 1:** Sociodemographic characteristics of the stroke cohort

	All poststroke patients	Patients with spasticity data^a^	Patients with spasticity^a^	Patients without spasticity^a^	*P* value*
N	460	328	54	274	
Age, mean ± SD, y	67.0 ± 14.0	65.8 ± 13.9	59.7 ± 14.1	67.1 ± 13.5	<.01
Female, *n* (%)	219 (47.6)	159 (48.5)	25 (46.0)	134 (49.0)	.73
Black, *n* (%)	113 (24.6)	84 (25.6)	21 (38.9)	63 (23.0)	.01
Residence at admission, *n* (%)					
Home	437 (95.0)	312 (95.1)	51 (94.4)	261 (95.3)	.73
Not home	23 (5.0)	16 (4.9)	3 (5.6)	13 (4.7)	
Marital status, *n* (%)					
Married/Partner	236 (51.3)	167 (50.9)	25 (46.3)	142 (51.8)	.46
Single	224 (48.7)	161 (49.1)	29 (53.7)	132 (48.2)	
Employment status, *n (%)*					< .01
Employed	131 (28.5)	97 (29.6)	18 (33.3)	79 (28.8)	
Unemployed/Retired/Other	290 (63.0)	199 (60.7)	24 (44.4)	175 (63.9)	
On disability	39 (8.5)	32 (9.8)	12 (22.2)	20 (7.3)	
Education					.93
<HS	153 (33.5)	102 (31.2)	18 (33.3)	84 (30.8)	
HS/GED test	127 (27.9)	93 (28.4)	15 (27.8)	78 (28.6)	
Some college	176 (38.6)	132 (40.4)	21 (38.9)	111 (40.7)	

Abbreviations: *GED* general education development, *HS* high school, *SD* standard deviation

^a^At 3 months

*Comparison between patients with and without spasticity

**Table 2:** Clinical characteristics of the stroke cohort

	All poststroke patients	Patients with spasticity data^a^	Patients with spasticity^a^	Patients without spasticity^a^	*P* value*
N	460	328	54	274	
BMI, median (IQR)	27.4 (24.2–31.5)	27.9 (24.6–32.3)	28.1 (24.4–33.8)	27.9 (24.6–31.7)	.50
Current smoker, *n* (%)	143 (31.0)	103 (31.0)	19 (35.0)	84 (31.0)	.51
Comorbidities, *n* (%)					
Cardiovascular	201 (43.7)	134 (41.0)	20 (37.0)	114 (41.6)	.53
Diabetes	164 (35.6)	115 (35.0)	21 (38.9)	94 (34.3)	.52
Depression (CESD ≥10)	180 (39.2)	125 (38.0)	24 (44.4)	101(36.9)	.29
Seizure	30 (6.5)	20 (6.0)	8 (14.8)	12 (4.4)	.01
Infection	82 (17.9)	54 (17.0)	8 (14.8)	46 (16.8)	.71
Prestroke Rankin (pre-mRS), median (IQR)	1 (0, 2)	1 (0, 2)	1 (0, 2)	1 (0, 2)	.68
Lesion volume, median (IQR)	*N* = 205	*N* = 143	*N* = 23	*N* = 120	.20
	2 (0–14)	2 (0–16)	2 (1–39)	2 (0–12)	
Estimated rNIHSS, median (IQR)	4 (2–7)	4 (2–7)	6 (3–10)	4 (2–6)	.02

Abbreviations: *BMI* body mass index, *CESD* Center for Epidemiologic Studies Depression scale, *IQR* interquartile range, *mRS* modified Rankin Scale, *rNIHSS* retrospective National Institute of Health Stroke Scale

^a^At 3 months

*Comparison between patients with and without spasticity

**Fig. 1:**
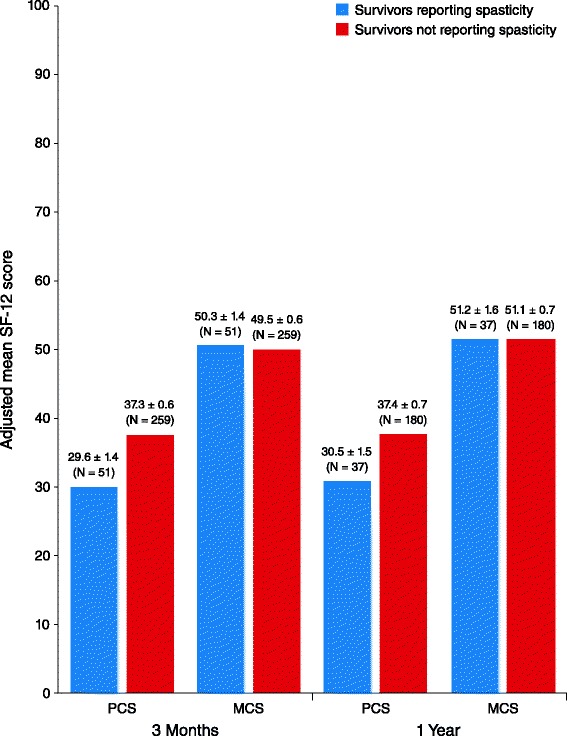
Adjusted mean Short Form-12 (SF-12) scores, by patient-reported spasticity presence. Significantly lower mean Physical Component Summary (PCS) and Mental Component Summary (MCS) scores were reported by survivors with spasticity than those without spasticity at 3 months (mean PCS difference, −7.7; 95 % confidence interval [CI] –10.7 to −4.6; *P* < .001 and mean MCS difference 0.8; 95 % CI, −2.2 to 3.8; *P* = .59) and at 1 year (mean PCS difference −6.9; 95 % CI −10.3 to −3.6; *P* < .001 and mean MCS difference 0.1; 95 % CI −3.3 to 3.5; *P* = .96) poststroke. The SF-12 was not administered at the 2-year interview. Scores were adjusted for age, race, stroke severity using the retrospective National Institute of Health Stroke Scale, prestroke function using the modified Rankin Scale, and comorbidities

**Fig. 2:**
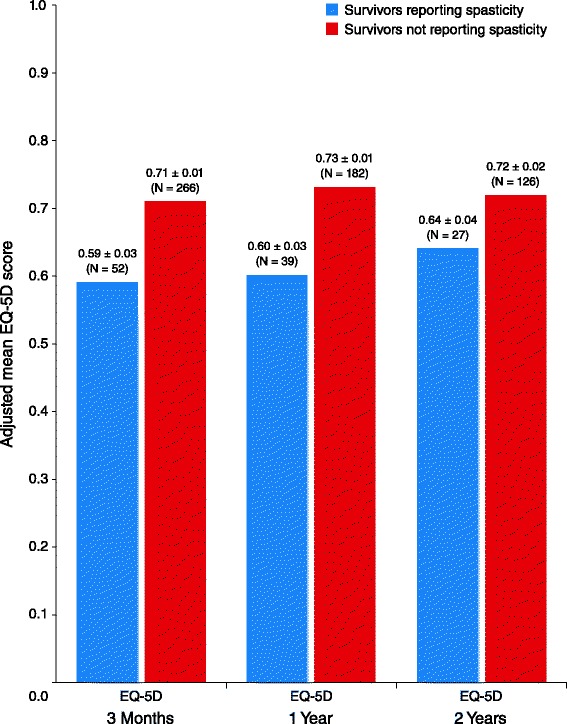
Adjusted mean EuroQol-5 dimension (EQ-5D) scores by patient-reported spasticity presence. Significantly lower EQ-5D scores were reported by survivors with spasticity than those without spasticity at 3 months (mean difference −0.12; 95 % confidence interval [CI] –0.18 to −0.06; *P* < .001), at 1 year (mean difference −0.12; 95 % CI −0.19 to −0.06; *P* < .001), and at 2 years (mean difference −0.08; 95 % CI −0.16 to −0.0004; *P* = .049) poststroke. Scores were adjusted for age, race, stroke severity using the retrospective National Institute of Health Stroke Scale, prestroke function using the modified Rankin Scale, and comorbidities

**Fig. 3:**
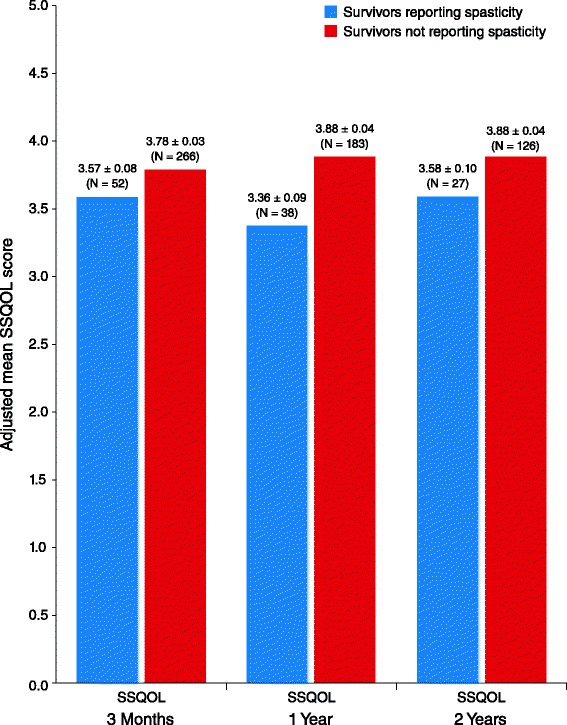
Adjusted mean Stroke-Specific Quality of Life (SSQOL) scores by patient-reported spasticity presence. Significantly lower SSQOL scores were reported by survivors with spasticity than those without spasticity at 3 months (mean difference −0.20; 95 % CI −0.38 to −0.02; *P* = .03), at 1 year (mean difference −0.51; 95 % CI −0.70 to −0.32; *P* < .001), and at 2 years (mean difference −0.30; 95 % CI −0.52 to −0.09; *P* = .01) poststroke. Scores were adjusted for age, race, stroke severity using the retrospective National Institute of Health Stroke Scale, prestroke function using the modified Rankin Scale, and comorbidities

The mean (± standard error [SE]) PCS score was lower for patients who reported spasticity at 3 months than for those who did not report spasticity (29.6 ± 1.4 vs 37.3 ± 0.6, respectively; mean difference −7.7; 95 % confidence interval [CI] [8] –10.7 to −4.6; *P* < .001). A significant difference between spasticity groups was not observed in 3-month mean MCS scores (50.3 ± 1.4 vs 49.5 ± 0.6; mean difference 0.8; 95 % CI −2.2 to 3.8; *P* = .59). Similar results were observed for PCS and MCS mean scores at 1 year (Fig. 1). Lower EQ-5D scores were reported by those with spasticity than without spasticity at 3 months (0.59 ± 0.03 vs 0.71 ± 0.01; mean difference −0.12; 95 % CI −0.18 to −0.06; *P* < .001) and at 1 and 2 years (Fig. 2). SSQOL scores were also lower in those with spasticity compared with those without spasticity at 3 months (3.57 ± 0.08 vs 3.78 ± 0.03; mean difference −0.20; 95 % CI −0.38 to −0.02; *P* = .03), as well as at 1 and 2 years (Fig. 3). In the longitudinal models, the effects of spasticity did not vary by followup time (i.e., the impact of spasticity on HRQoL did not vary significantly from 3 months to 1 year or 1 year to 2 years). Therefore, an average effect of spasticity over the followup time is presented in Table 3.

**Table 3:** Poststroke adjusted mean HRQoL scores by spasticity reporting

HRQoL	Patients with spasticity,	Patients without spasticity,	Mean difference (95 % CI)^a^
	mean ± SE	mean ± SE	
	(95 % CI)	(95 % CI)	
SF-12			
Physical Component Summary	31.1 ± 0.9	37.0 ± 0.5	−5.8 (−7.8 to −4.0)
	(29.4–32.9)	(36.0–38.0)	
Mental Component Summary	50.7 ± 1.3	50.11 ± 0.5	0.6 (−2.1 to 3.2)
	(48.2–53.2)	(49.2–51.0)	
EQ-5D			
Index score (US weighted)	0.63 ± 0.02	0.71 ± 0.01	−0.07 (−0.12 to −0.03)
	(0.59–0.68)	(0.69–0.73)	
SSQOL			
Overall score	3.6 ± 0.05	3.8 ± 0.03	−0.21 (–0.32 to–0.11)
	(3.5–3.7)	(3.8–3.9)	

Abbreviations: *CI* confidence interval, *EQ-5D* EuroQol-5 dimension, *HRQoL* health-related quality of life, *SF-12* Short Form-12, *SE* standard error, *SSQOL* Stroke-Specific Quality of Life, and *US* United States

^a^Adjusted for age, race, and retrospective National Institute of Health Stroke Scale

## Discussion

Stroke causes a decrease in HRQoL with multiple factors, such as age, gender, dependency in activities of daily living, social support, depression, institutionalization, and diabetes associated with poorer HRQoL in stroke survivors [19]. The results of this study demonstrate that the presence of spasticity also independently impacts the HRQoL of stroke survivors. Stroke survivors with spasticity were associated with lower HRQoL compared with stroke survivors without spasticity.

Although minimal clinically important differences (MCID; the smallest difference in score that would be considered meaningful by a patient) for the EQ-5D, SF-12, and SSQOL have not been established in the stroke population, the differences in EQ-5D scores observed in this study between stroke survivors with spasticity and without spasticity met MCIDs established in other diseases. For example, Walters and Brazier estimated the average MCID of the EQ-5D to be 0.07 across studies that included patients with leg ulcers, back pain, rheumatoid arthritis, osteoarthritis, irritable bowel, acute myocardial infarction, and chronic obstructive pulmonary disease, and Le et al. recently estimated the MCID of the EQ-5D to range from 0.3 to 0.1 in patients with posttraumatic stress disorder [20, 21]. In our study, the mean EQ-5D differences between patients with and without spasticity at 3 months, 1 year, and 2 years were 0.12, 0.12, and 0.08, respectively. To clinicians who manage stroke survivors with spasticity, these meaningful differences likely do not come as a surprise given the day-to-day effect that spasticity has on individuals suffering from this condition. When individuals with spasticity have been asked which single aspect of spasticity has the greatest impact on their HRQoL, the most common answers were limited range of motion, stiffness or contracture of muscles, and limitations in activities of daily living [7]. To our knowledge, the results of this study are the first to quantify the impact of poststroke spasticity on HRQoL.

The implications of these results are 3-fold. First, spasticity must be prevented if at all possible and identified once presenting in stroke survivors. Unfortunately, little is known about the predictors of spasticity and a simple measure to identify spasticity does not exist. Future work to understand the predictors of spasticity and improve the identification of spasticity is needed, specifically in the community setting. Second, once identified, a stroke survivor with spasticity should be assessed for treatment or be referred to a physician educated in spasticity management. Last, if treatment is required, a multidisciplinary approach needs to be taken in order for the patient to get the most benefit from existing treatments. Potential treatments include physical rehabilitation, oral medications, botulinum toxin A, phenol, and surgery.

There are limitations to this study. The findings may not be generalizable to all ischemic stroke patients since study participates had to survive their stroke hospitalization and reside in the Greater Cincinnati/Northern Kentucky region. The prevalence of spasticity in the cohort was 16 % at 3 months poststroke, lower than rates reported in other studies. This may be in part due to spasticity being patient-reported, which may have led to the classification of stoke survivors as nonspastic when indeed spasticity was present. Also, the study did not examine current use of spasticity medication and did not include a spasticity-specific HRQoL measure. The validation of the shortened version of the stroke-specific instrument (SSQOL) used in this study has not been published. As is the case for all observational studies, all confounding factors may not have been controlled for, but a diligent attempt was made to adjust for known confounders, such as age, race, stroke severity, pre-stroke function, and comorbidities.

## Conclusions

Spasticity is associated with a negative impact on the HRQoL of stroke survivors with statistically and clinically meaningful differences existing between stroke survivors with and without spasticity. These results suggest an opportunity to improve HRQoL among stroke survivors with spasticity.

## Abbreviations

CI: confidence interval
EQ-5D: EuroQol-5 dimension
GCNKSS: Greater Cincinnati/Northern Kentucky Stroke Study
HRQoL: health-related quality of life
MCID: minimal clinically important differences
MCS: Mental Component Summary
mRS: modified Rankin Scale
PCS: Physical Component Summary
rNIHSS: retrospective National Institutes of Health Stroke Scale
SE: standard error
SF-12: Short Form-12
SSQOL: Stroke-Specific Quality of Life
